# Supplementary material for: PRPS activity tunes redox homeostasis in Myc-driven lymphoma
Source: bioRxiv. 2025 Jan 13:2025.01.08.632009. Preprint. [Version 1] doi: 10.1101/2025.01.08.632009 (PMC11761749; doi:10.1101/2025.01.08.632009)
Supplement: Supplementary file 1 [file NIHPP2025.01.08.632009v1-supplement-3.pdf]

bioRxiv preprint doi: https://doi.org/10.1101/2025.01.08.632009; this version posted January 13, 2025. The copyright holder for this preprint (which was not certified by peer review) is the author/funder, who has granted bioRxiv a license to display the preprint in perpetuity. It is made available under aCC-BY 4.0 International license.

Supplementary Figure 1

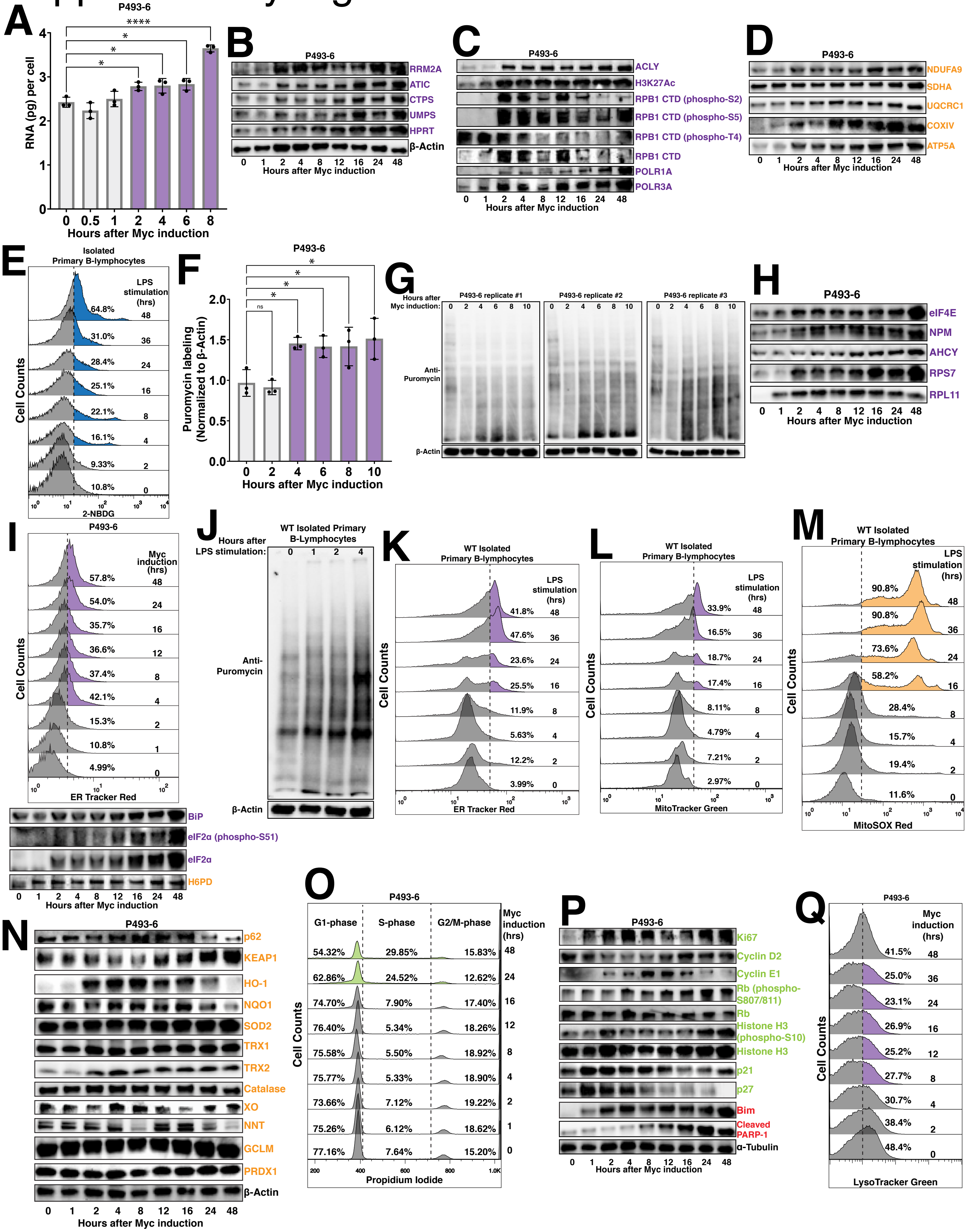

bioRxiv preprint doi: https://doi.org/10.1101/2025.01.08.632009; this version posted January 13, 2025. The copyright holder for this preprint (which was not certified by peer review) is the author/funder, who has granted bioRxiv a license to display the preprint in perpetuity. It is made available under aCC-BY 4.0 International license.

Supplementary Figure 1 – Related to Figure 1

(A) RNA content per cell measured in P493-6 cells over an 8hr time course following tetracycline removal to induce Myc expression.

Western blot of (B) nucleotide biosynthesis gene expression, (C) transcriptional regulation and (D) mitochondrial complex gene expression in P493-6 cells over a 48hr time course following tetracycline removal to induce Myc expression. β-Actin used as a loading control.

(E) Glucose uptake in WT murine primary B lymphocytes over a 48hr time course following LPS stimulation, measured via 2-NBDG.

(F,G) Puromycylation assay measuring protein synthesis in P493-6 peptides over a 10hr time course following tetracycline removal to induce Myc expression, normalized to β-Actin.

(H) Western blot of translational regulation in P493-6 cells over a 48hr time course following tetracycline removal to induce Myc expression.

(I) Endoplasmic reticulum (ER) expansion in P493-6 cells over a 48hr time course following tetracycline removal to induce Myc expression, measured via ER Tracker Red (top). Western blot of ER-localized and ER-stress response protein expression in P493-6 cells over a 48hr time course following tetracycline removal to induce Myc expression (bottom).

(J) Puromycylation assay measuring protein synthesis in WT murine primary B lymphocyte peptides over a 4hr time course following LPS stimulation. β-Actin used as a loading control.

(K) ER expansion, measured via ER Tracker Red and (L) mitochondrial mass, measured via MitoTracker Green in WT murine primary B lymphocytes over a 48hr time course following LPS stimulation.

(M) Mitochondrial ROS accumulation in WT murine primary B lymphocytes over a 48hr time course following LPS stimulation, measured via MitoSOX Red.

(N) Western blot analysis of antioxidant response element (ARE) metabolic enzyme expression in P493-6 cells over a 48hr time course following tetracycline removal to induce Myc expression. β-Actin used as a loading control.

(O) Cell cycle analysis of P493-6 cells over a 48hr time course following tetracycline removal to induce Myc expression, measured via Propidium Iodide. Cell cycle profiling quantified as a percentage of cells in G1, S and G2/M phases at each time point.

(P) Western blot of cell cycle regulation and apoptosis in P493-6 cells over a 48hr time course following tetracycline removal to induce Myc expression. α-Tubulin used as a loading control.

(Q) Lysosomal content in P493-6 cells over a 48hr time course following tetracycline removal to induce Myc expression, measured via LysoTracker Green.

For all panels, statistical analysis performed via One-Way ANOVA, bars represent mean ± s.d.; *p<0.05, ****p<0.0001, ns: not significant. For all histograms, upregulation/downregulation quantified as a percentage of the population to the right of the dashed line at each time point.

bioRxiv preprint doi: https://doi.org/10.1101/2025.01.08.632009; this version posted January 13, 2025. The copyright holder for this preprint (which was not certified by peer review) is the author/funder, who has granted bioRxiv a license to display the preprint in perpetuity. It is made available under aCC-BY 4.0 International license.

Supplementary Figure 2

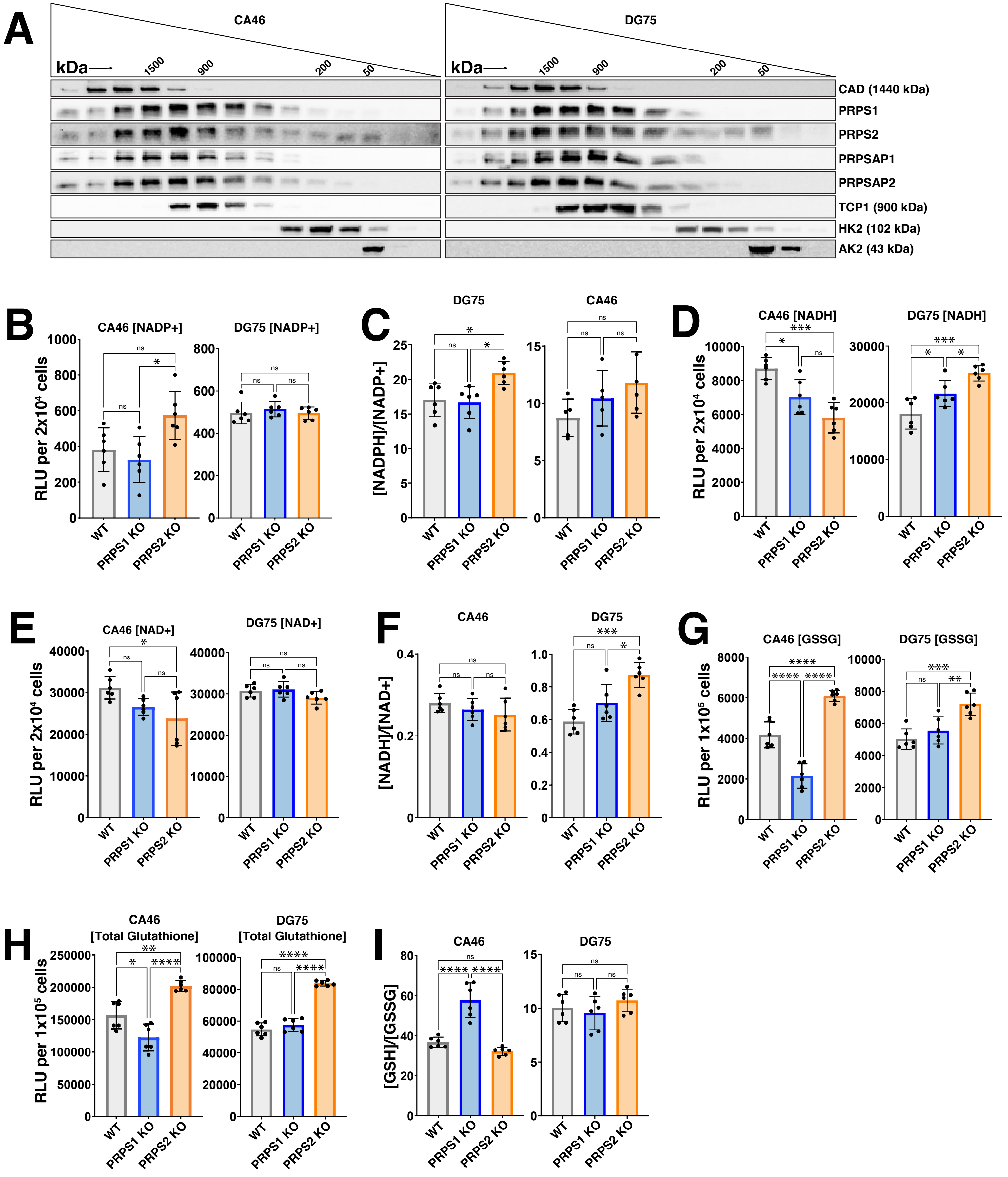

bioRxiv preprint doi: https://doi.org/10.1101/2025.01.08.632009; this version posted January 13, 2025. The copyright holder for this preprint (which was not certified by peer review) is the author/funder, who has granted bioRxiv a license to display the preprint in perpetuity. It is made available under aCC-BY 4.0 International license.

Supplementary Figure 2 – Related to Figure 2 and Figure 3
(A) Western blot analysis of PRPS complex coordination using fractions collected from size exclusion chromatography (SEC) runs to visualize PRPS complex size in the context of validated internal standards (CAD, TCP-1η, HK2, AK2) in CA46 (left) and DG75 (right) cell lines. (B) NADP+ levels, (C) [NADPH]/[NADP+] ratio, (D) NADH levels, (E) NAD+ levels, (F) [NADH]/[NAD+] ratio, (G) oxidized glutathione (GSSG) levels, (H) total glutathione levels and (I) [GSH]/[GSSG] ratio in WT, PRPS1- and PRPS2- KO cells of CA46 (left) and DG75 (right) cell lines, measured via relative luciferase units (RLU) of luminescent-based GLO-assays.
in WT, PRPS1- and PRPS2- KO cells of CA46 (left) and DG75 (right) cell lines.

For all panels, statistical analysis performed via One-Way ANOVA, bars represent mean ± s.d.; *p<0.05, **p<0.01, ***p<0.001, ****p<0.0001, ns: not significant.

bioRxiv preprint doi: https://doi.org/10.1101/2025.01.08.632009; this version posted January 13, 2025. The copyright holder for this preprint (which was not certified by peer review) is the author/funder, who has granted bioRxiv a license to display the preprint in perpetuity. It is made available under aCC-BY 4.0 International license.

Supplementary Figure 3

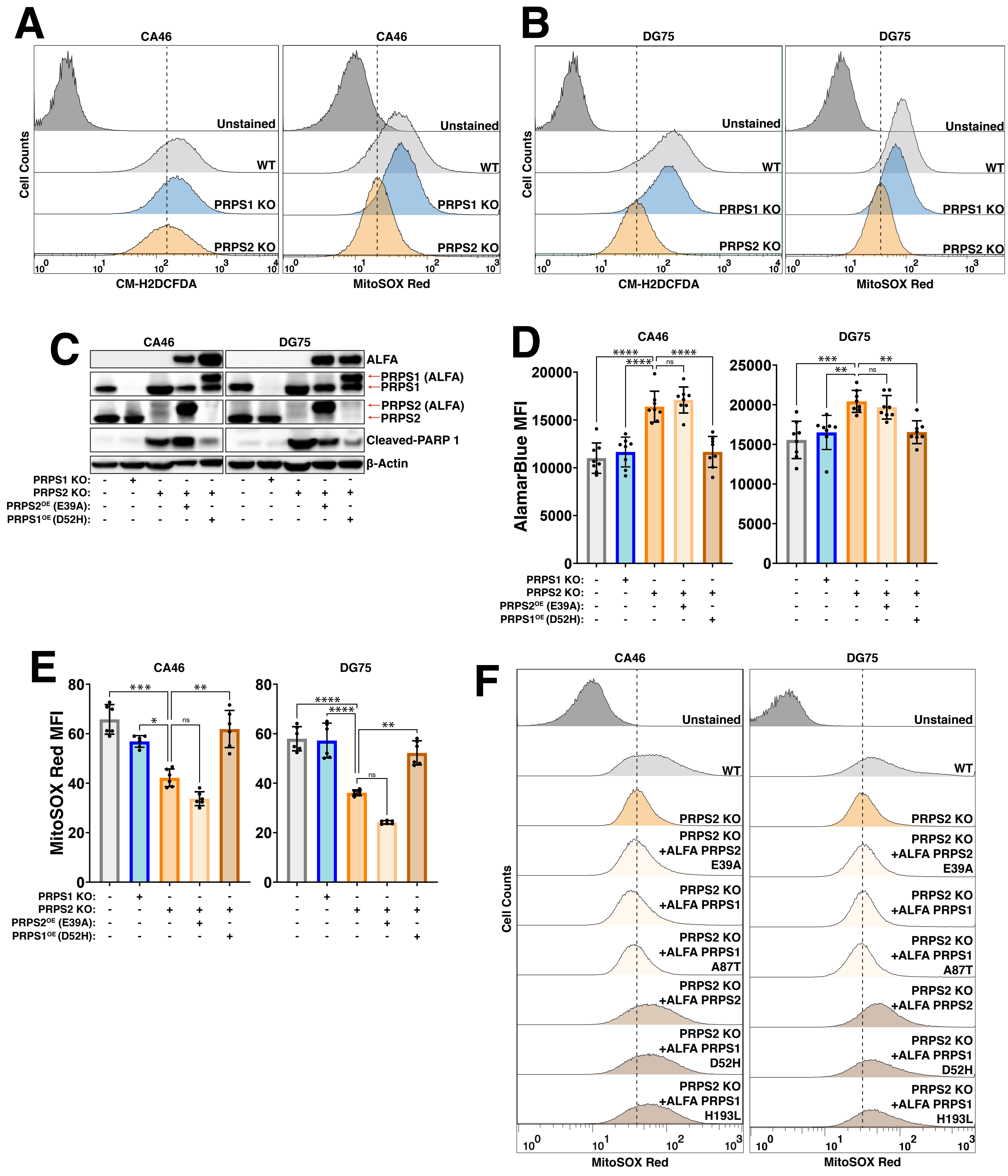

bioRxiv preprint doi: https://doi.org/10.1101/2025.01.08.632009; this version posted January 13, 2025. The copyright holder for this preprint (which was not certified by peer review) is the author/funder, who has granted bioRxiv a license to display the preprint in perpetuity. It is made available under aCC-BY 4.0 International license.

Supplementary Figure 3 – Related to Figure 3
ROS accumulation in WT, PRPS1- and PRPS2- KO cells of (A) CA46 and (B) DG75 cell lines, measured via the total intracellular CM-H_2DCFDA (left) and mitochondrial-specific MitoSOX Red (right) dyes.
(C) Western blot validating stable exogenous expression of ALFA-tagged PRPS1 superactive (D52H) and PRPS2 catalytically inactive (E39A) mutant constructs in CA46 (left) and DG75 (right) PRPS2 KO cell lines. 24 kDa PARP-1 fragment is used as an apoptotic marker. β-Actin used as a loading control.
(D) AlamarBlue mean fluorescence intensity (MFI) as a readout of intracellular reduction and (E) MitoSOX Red MFI as a readout of mitochondrial ROS accumulation in WT, PRPS1 KO, PRPS2 KO and PRPS2 KO cells containing stably integrated ALFA-tagged PRPS1 superactive mutant (D52H) and PRPS2 catalytically inactive mutant (E39A) constructs in CA46 (left) and DG75 (right) cell lines.
(F) Mitochondrial ROS accumulation in WT, PRPS1 KO, PRPS2 KO and PRPS2 KO cells stably integrated with ALFA-tagged PRPS1, PRPS2, PRPS1 hypomorphic mutant (A87T), PRPS1 superactive mutant (D52H, H193L) and PRPS2 catalytically inactive mutant (E39A) constructs in CA46 (left) and DG75 (right) cell lines, measured via MitoSOX Red.

For all panels, statistical analysis performed via One-Way ANOVA, bars represent mean ± s.d.; *p<0.05, **p<0.01, ***p<0.001, ****p<0.0001, ns: not significant. For all histograms, dashed lines indicate MFI of PRPS2 KO cells.

bioRxiv preprint doi: https://doi.org/10.1101/2025.01.08.632009; this version posted January 13, 2025. The copyright holder for this preprint (which was not certified by peer review) is the author/funder, who has granted bioRxiv a license to display the preprint in perpetuity. It is made available under aCC-BY 4.0 International license.

Supplementary Figure 4

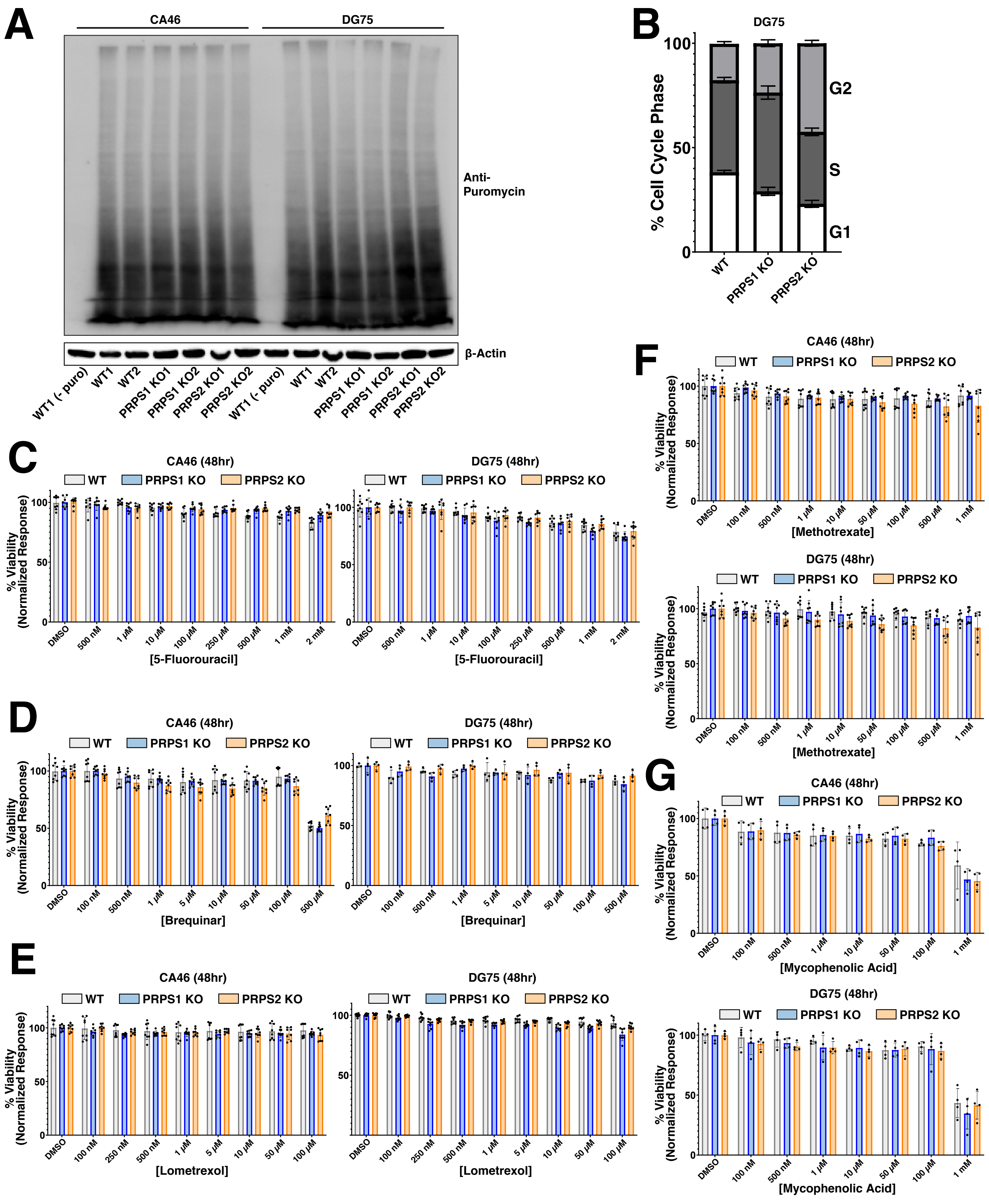

bioRxiv preprint doi: https://doi.org/10.1101/2025.01.08.632009; this version posted January 13, 2025. The copyright holder for this preprint (which was not certified by peer review) is the author/funder, who has granted bioRxiv a license to display the preprint in perpetuity. It is made available under aCC-BY 4.0 International license.

Supplementary Figure 4 – Related to Figure 4

(A) Puromycylation assay measuring protein synthesis in WT, PRPS1- and PRPS2- KO cells of CA46 and DG75 cell lines. Each cell line is represented with two independent clones, per genotype. β-Actin used as a loading control.

(B) Cell cycle analysis profiling the percentage of cells in G1, S, and G2 phases for WT, PRPS1- and PRPS2- KO cells of DG75 cells.

(C-G) Viability response of individual replicates of WT, PRPS1- and PRPS2- KO cells of CA46 (left/top) and DG75 (right/bottom) cell lines to treatment with increasing concentrations of (C) 5-fluorouracil, (D) brequinar, (E) lometrexol, (F) methotrexate and (G) mycophenolic acid, normalized to vehicle treatment.

For all panels, bars represent mean ± s.d.

bioRxiv preprint doi: https://doi.org/10.1101/2025.01.08.632009; this version posted January 13, 2025. The copyright holder for this preprint (which was not certified by peer review) is the author/funder, who has granted bioRxiv a license to display the preprint in perpetuity. It is made available under aCC-BY 4.0 International license.

Supplementary Figure 5

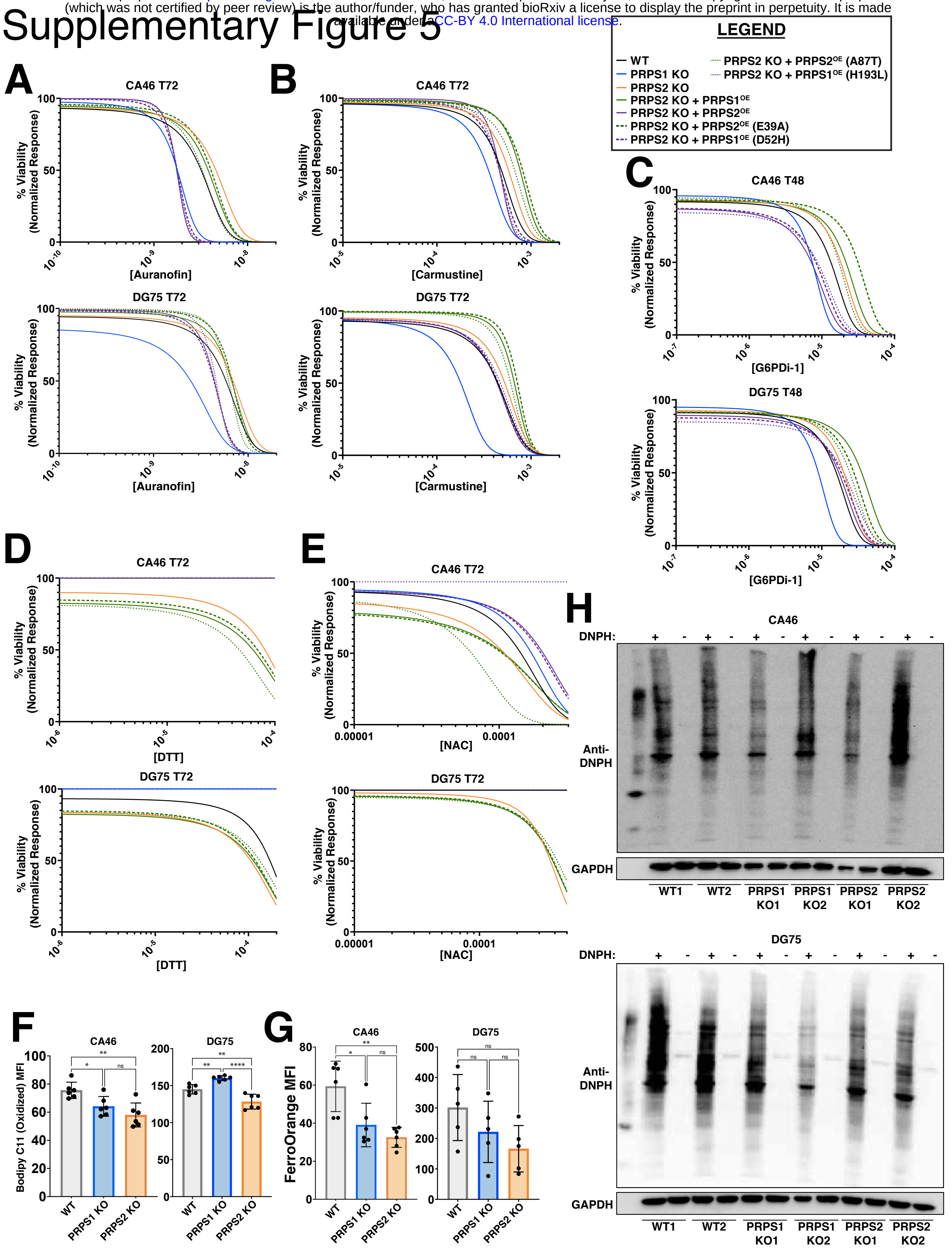

bioRxiv preprint doi: https://doi.org/10.1101/2025.01.08.632009; this version posted January 13, 2025. The copyright holder for this preprint (which was not certified by peer review) is the author/funder, who has granted bioRxiv a license to display the preprint in perpetuity. It is made available under aCC-BY 4.0 International license.

Supplementary Figure 5 – Related to Figure 5
(A-E) Dose-response curves illustrating viability responses to (A) auranofin, (B) carmustine, (C) G6PDi-1, (D) DTT and (E) NAC treatment in WT, PRPS1 KO, PRPS2 KO and PRPS2 KO cells stably integrated with ALFA-tagged PRPS1, PRPS2, PRPS1 hypomorphic mutant (A87T), PRPS1 superactive mutants (D52H, H193L) and PRPS2 catalytically inactive mutant (E39A) constructs in CA46 (top) and DG75 (bottom) cell lines, normalized to vehicle treatment. X-axis represents the logarithmic scale of increasing drug concentration, Y-axis represents the normalized response as a viability percentage. Time points are indicated on each individual graph, determined by R^2 values for goodness-of-fit (Data represented as a mean of normalized response of individual replicates at each concentration tested).
(F) Lipid peroxidation, measured via BODIPY C11 mean fluorescence intensity (MFI) and (G) labile intracellular iron, measured via FerroOrange MFI in WT, PRPS1- and PRPS2- KO cells of CA46 (left) and DG75 (right) cell lines.
(H) Western blot illustrating levels of global protein oxidation, via carbonyl side chain derivatization by 2,4-dinitrophenylhydrazine (DNPH), in WT, PRPS1- and PRPS2- KO cells of CA46 (top) and DG75 (bottom cell lines). (-) DNPH lanes serve as a control for DNPH-mediated derivatization. GAPDH is used as a loading control.

For all panels, statistical analysis performed via One-Way ANOVA, bars represent mean ± s.d.; *p<0.05, **p<0.01, ****p<0.0001, ns: not significant.
